# Design Optimization and Mechanical Performance Evaluation of a Modified Coronary IV-OCT Catheter Adapted for Intracranial Navigation: A Preclinical Study

**DOI:** 10.3390/bios15110755

**Published:** 2025-11-12

**Authors:** Tahsin Nairuz, Young-Suk Hwang, Min-Yong Kwon, Jae Hyun Kim, Sae Min Kwon, Hyuck-Jun Yoon, Seung-Ho Hur, Joonho Chung, Woo-Jin Kim, Sang-Hyun An, Jun Sik Kim, Jong-Ha Lee, Chang-Hyun Kim

**Affiliations:** 1 Department of Biomedical Engineering, Keimyung University, Daegu 42601, Republic of Korea; tahsin.bmb@nstu.edu.bd; 2 Department of Neurosurgery, Keimyung University School of Medicine, Daegu 42601, Republic of Korea; dh86881@gmail.com (Y.-S.H.); only1back@me.com (M.-Y.K.); dhggo@naver.com (J.H.K.); kwonsaemin@hanmail.net (S.M.K.); 3 Department of Cardiology, Keimyung University School of Medicine, Daegu 42601, Republic of Korea; hippsons@dsmc.or.kr (H.-J.Y.); shur@dsmc.or.kr (S.-H.H.); 4 Department of Neurosurgery, Yonsei University College of Medicine, Seoul 6273, Republic of Korea; ns.joonho.chung@gmail.com; 5 Biomedical Manufacturing Technology Center (BMTC), Korea Institute of Industrial Technology (KITECH), Yeongcheon 38822, Republic of Korea; woojinkim@kitech.re.kr; 6 Preclinical Research Center, Daegu-Gyeongbuk Medical Innovation Foundation (KMedi Hub), Daegu 41061, Republic of Korea; ash4235@kmedihub.re.kr (S.-H.A.); jskim@kmedihub.re.kr (J.S.K.)

**Keywords:** intravascular optical coherence tomography, neurovascular interventions, catheter modification, mechanical performance, trackability and pushability

## Abstract

The application of intravascular optical coherence tomography (IV-OCT) in neurovascular interventions is constrained by the mechanical inadequacy of conventional catheters in navigating the complex intracranial vasculature. To address this, we modified a coronary IV-OCT catheter, enhancing its mechanical performance for neurovascular applications. The modified catheter featured a 300 mm over-the-microwire segment and a dual-structured shaft (distal 50 mm nonbraided, proximal 250 mm braided) to improve trackability and pushability. We compared the modified and conventional catheters using a benchtop model with a simulated vessel path and an in vivo swine model. Trackability and pushability were quantitatively measured using resistance (N) and advancement distance (mm) in the simulated path. In the animal model, indirect performance metrics included the catheter tension angle (CTA) and pass of catheter (POC) through the fourth curvature of the external carotid artery (ECA). The modified catheter demonstrated superior pushability (172.9 ± 1.96 mm vs. 127.9 ± 2.86 mm, *p* < 0.05) and increased resistance (1.47 ± 0.036 N vs. 0.69 ± 0.032 N, *p* < 0.05). In vivo analysis further showed a significantly greater CTA (115.8 ± 8.5° vs. 77.6 ± 10.3°, *p* < 0.05) and higher POC success rate (83.3% vs. 11.1%, *p* < 0.05). These results indicate that the modified coronary IV-OCT catheter offers enhanced mechanical performance, suggesting its potential for safe and effective use in neurovascular procedures.

## 1. Introduction

Advancements in minimally invasive therapeutic technologies have markedly transformed vascular intervention; however, the diagnosis and management of intracranial arterial disorders and aneurysms remain hindered by the limitations of current imaging modalities. Conventional non-invasive techniques often lack the spatial resolution required to accurately visualize key anatomical features such as the arterial wall pathology, small perforating branches, and the interface between vascular devices and native tissue. These shortcomings also hinder the assessment of device-related complications, including platelet aggregation and malapposition. While intravascular imaging tools—such as those employed in coronary and peripheral arteries—offer improved visualization, they are not yet optimized for the intricate and tortuous anatomy of the cerebrovascular system. Accurate diagnosis is further impeded by the intrinsic limitations of standard imaging techniques, which suffer from suboptimal contrast resolution, susceptibility to metallic artifacts, and reduced visibility in miniaturized, low-profile neurovascular devices [1,2]. As devices have evolved to become more deliverable within tortuous intracranial vessels, their radiographic visibility has paradoxically diminished, complicating procedural decision-making and post-treatment assessment.

To overcome these obstacles, there is a compelling need for an intravascular imaging modality that can simultaneously visualize arterial tissue and implanted devices with micrometer-scale resolution. Intravascular optical coherence tomography (IV-OCT) represents a powerful candidate modality in this regard. Utilizing low-coherence interferometry, IV-OCT generates high-resolution cross-sectional images based on the backscattering properties of biological tissues, enabling in situ volumetric microscopy with exceptional spatial resolution, dynamic range, and contrast sensitivity [3]. In the cardiovascular field, IV-OCT has become a transformative diagnostic and research tool, significantly enhancing our understanding of lumen morphology, stent-vessel interactions, and real-time vascular responses to therapeutic interventions [4,5,6,7]. While extensively utilized in cardiovascular practice, the translational potential of intravascular OCT into the neurovascular domain holds promise for overcoming longstanding visualization limitations that hinder both the efficacy and safety of intracranial endovascular procedures [8,9].

Nonetheless, contemporary intravascular imaging catheters remain fundamentally inadequate for routine use within the highly tortuous and anatomically constrained intracranial vasculature of the human brain [8]. These devices are often mechanically incompatible with the low-profile neurovascular microcatheters routinely employed in endovascular procedures. Specifically, the increased shaft stiffness and limited over-the-wire segment impede advancement through sharply angulated vessels, particularly in the anterior circulation. For example, the monorail Dragonfly IV-OCT catheter (St. Jude Medical, St. Paul, MN, USA), while widely adopted in cardiovascular practice, exhibits a short over-the-wire configuration and high shaft flexibility—features that hinder its navigability in the complex intracranial anatomy [10,11,12,13]. Additionally, the design of existing catheters fails to ensure rotational stability or mitigate imaging artifacts under conditions of vascular tortuosity, frequently resulting in distorted cross-sectional reconstructions and reduced interpretability of acquired data [14]. Consequently, the clinical application of coronary imaging systems has been largely confined to the proximal, relatively straight segments of the intracranial circulation, where anatomical complexity is minimal [11,15,16,17]. Moreover, the restricted imaging field of view in current catheter designs precludes comprehensive visualization of large-diameter intracranial vessels, aneurysmal structures, and the adjacent subarachnoid space—limiting their diagnostic utility in evaluating both the intraluminal and perivascular environments.

Recognizing the diagnostic potential of IV-OCT in cerebrovascular disease, several studies have explored neuro-specific adaptations of the technology [18]. A previous report described the creation of brain-applicable OCT catheters for investigating the feasibility of neuroendovascular OCT and comparing the neuroendovascular OCT findings with those of histology in nondiseased vessels in animal, cadaveric, and clinical studies [19]. A recently published paper also presented a neurovascular high-frequency OCT system with an imaging and endoscopic probe designed in tortuous cerebrovascular segments [20]. However, most of these designs remain at the conceptual or early experimental stage, and a catheter system that combines sufficient pushability and trackability for routine clinical navigation through tortuous intracranial pathways has not yet been achieved.

To address this gap, previously we developed a cerebrovascular-specific optical coherence tomography (bOCT) catheter, representing a structural evolution of the coronary OCT platform to overcome the mechanical limitations imposed by tortuous neurovascular anatomy [18]. Extending that line of innovation, we developed a structurally modified catheter, designated as the cranial IV-OCT catheter to overcome the limitations of conventional coronary systems and to apply the catheter in the curved intracranial vessels. This version incorporates dimensional refinements, optimized shaft stiffness gradients, and a reconfigured distal tip to enable safer, more stable navigation through the sharply angulated intracranial vasculature while maintaining imaging fidelity. The catheter’s mechanical feasibility and navigational performance were quantitatively evaluated to assess its potential for future neurovascular OCT applications.

## 2. Materials and Methods

### 2.1. Design Rationale and Structural Modifications of Coronary IV-OCT Catheter for Neurovascular Applications

**Figure 1:**
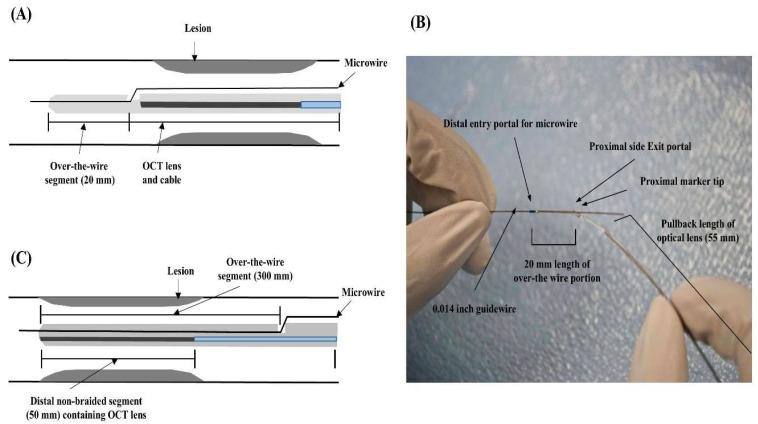
(**A**) Schematic of the conventional coronary IV-OCT catheter showing the lesion site, microwire, short over-the-wire segment (20 mm) located distal to the lesion, and the OCT lens with connecting cable. (**B**) Actual image of conventional coronary IV-OCT catheter. (**C**) Schematic of the modified cranial IV-OCT catheter showing the lesion site, microwire, elongated over-the-wire segment (300 mm) extending from distal entry to proximal exit, and the dual-structured shaft consisting of a distal non-braided segment (50 mm) containing the OCT lens and a proximal braided segment (250 mm) designed to enhance pushability.

The proposed cranial IV-OCT catheter was conceptualized as a structurally modified alternative to the conventional coronary IV-OCT system (C7 Dragonfly imaging catheter, St. Jude Medical, St. Paul, MN, USA), with the specific objective of facilitating traversal through intricate or severely stenotic intracranial arteries. The pronounced flexibility of the conventional coronary IV-OCT catheter, while effective in coronary applications, limits its utility in navigating such challenging vascular anatomies. Structural assessment revealed several critical design constraints: (1) the catheter advances over a microwire with a relatively short distal segment (~20 mm) extending beyond the wire; (2) the absence of a braided shaft significantly compromises pushability and force transmission along tortuous paths; and (3) the OCT lens is positioned proximal to the distal catheter tip, causing the tip to extend beyond the lesion site during imaging. As a result, the catheter frequently overshoots the target lesion, leading to suboptimal image acquisition and diminished procedural accuracy (Figure 1A,B).

To overcome these limitations, the modified cranial IV-OCT catheter was designed with targeted structural enhancements aimed at significantly improving pushability and trackability in curved intracranial vasculature. First, the over-the-microwire segment—extending from the distal entry to the proximal exit port—was lengthened to approximately 300 mm, a substantial increase from the ~20 mm seen in conventional coronary IV-OCT systems, thereby augmenting the catheter’s distal control and axial force transmission. Second, to minimize the overall catheter diameter and streamline intraluminal passage, the microwire lumen and the OCT lens connected to the optical fiber were designed without internal separation by a structural septum. Furthermore, the distal 300 mm of the catheter incorporated a dual configuration: the distal 50 mm was intentionally non-braided to avoid imaging interference from metallic coil artifacts, while the proximal 250 mm employed a braided design to augment pushability. To further mitigate the increased resistance typically associated with braided structures, a hydrophilic coating was applied to the surface of the catheter, thereby compensating for potential reductions in trackability (Figure 1C).

Moreover, in comparison with the bOCT catheter described in our previous work [18], the present design introduces several refinements aimed at improving adaptability to complex cerebrovascular geometries. The adaptation from the previous bOCT design was essential because the mechanical and anatomical demands of intracranial vessels are considerably different. Intracranial arteries are smaller in diameter and more fragile and exhibit sharper angulations, requiring a catheter with enhanced flexibility and precise force transmission to prevent vessel trauma during navigation. In the earlier version, the distal non-braided segment measured 30 mm, which was suitable for larger vessels but limited maneuverability in tortuous intracranial paths. In the current cranial IV-OCT catheter, this segment was extended to 50 mm, providing improved distal flexibility and reducing metallic interference near the optical lens. Additionally, the shaft composition and dual-lumen configuration were optimized to maintain axial strength while minimizing frictional resistance, thereby enhancing both pushability and imaging stability. These targeted modifications collectively strengthen the catheter’s suitability for potential intracranial navigation and high-fidelity OCT imaging applications. The descriptions and structural details of each part of the OCT catheter, including the inner and outer diameters of each layer, are provided in the Supplementary Files (Figures S1 and S2).

Based on this design rationale, the catheter was fabricated using established manufacturing techniques adapted to the new structural configuration. The elongated over-the-wire segment (~300 mm) was produced by precision extrusion of a polymer tube incorporating a 0.014-inch microwire lumen without a septum. The proximal 250 mm shaft was reinforced with a stainless-steel braid applied by standard braiding machinery, while the distal 50 mm segment was left non-braided to preserve OCT imaging quality. The catheter tip was thermally bonded to secure the optical fiber and lens assembly, and all joints were sealed using biocompatible medical-grade adhesives under cleanroom conditions. A hydrophilic polymer coating was subsequently applied to the external surface to reduce friction. Importantly, all fabrication steps (extrusion, braiding, bonding, coating) align with conventional catheter mass-production methods, suggesting scalability, although further process optimization, quality assurance, and regulatory validation will be required for clinical translation.

### 2.2. Evaluation of the Mechanical Performance of the Modified Cranial IV-OCT Catheter

To evaluate and compare the mechanical performance of the modified cranial IV-OCT catheter with the existing coronary IV-OCT catheter, a benchtop experimental setup was employed using a simulated blood vessel flow path (ASTM F2394) [21], designed to replicate the anatomical complexity of tortuous vessels. Among various mechanical parameters, trackability and pushability were selected as the primary performance indicators due to their clinical relevance in navigating tortuous intracranial vasculature. In this context, trackability was operationally defined as the maximum resistance (measured in Newtons, N) encountered by the catheter during advancement through the simulated vascular path, while pushability referred to the total distance (measured in millimeters, mm) achieved under continuous proximal force, reflecting the catheter’s capacity to transmit axial force efficiently to the distal tip—an essential factor in assessing the entry performance of a catheter in tortuous vascular pathways. Both resistance and distance metrics were directly measured to determine these properties, with increased resistance values corresponding to reduced trackability. Quantitative measurements were obtained using a force sensor integrated with a load cell for real-time resistance recording. During each trial, the catheter was advanced over a 0.014-inch guidewire under continuous proximal force, and the load cell captured the real-time axial resistance generated as the catheter navigated the tortuous path. The peak resistance value recorded was defined as the measure of trackability, while the corresponding advancement distance was simultaneously recorded to assess pushability. The experimental setup utilized distilled water in place of blood to maintain controlled conditions, and a 0.014-inch guidewire was employed to ensure consistent catheter alignment. The simulated path was set to a 240 mm length to encompass full catheter advancement and to observe mechanical deformation phenomena such as kinking. When kinking of the catheter was observed, both the resistance force and the corresponding travel distance were recorded.

### 2.3. Evaluation of the Modified Cranial IV-OCT Catheter for Traversing Curved Vessels in Animal Model

All animal procedures were conducted in accordance with protocols approved by the Institutional Animal Care and Use Committee of the Daegu-Gyeongbuk Medical Innovation Foundation (Approval No. DGMIF-20012201-03). The study adhered to the ARRIVE (Animal Research: Reporting of In Vivo Experiments) guidelines, with all efforts made to minimize animal usage and procedural distress. For this experiment, two 12-month-old female swine (Large White × Landrace × Duroc), each weighing between 30 and 40 kg, were selected. The swine were housed in facilities accredited by the Daegu-Gyeongbuk Medical Innovation Foundation and the Association for Assessment and Accreditation of Laboratory Animal Care (AAALAC) and were acclimated for a minimum of five days prior to experimentation. Animals were provided unrestricted access to food and water until the night preceding the procedure. Premedication consisting of 100 mg aspirin and 75 mg clopidogrel was administered daily for five consecutive days before the procedure.

On the day of the experiment, anesthesia was induced with an intramuscular injection of zolazepam (5 mg/kg; Zoletil 50, Virbac Korea, Seoul, Republic of Korea) and xylazine hydrochloride (2 mg/kg; Rompun, Bayer, Leverkusen, Germany) following a 12 h fasting and 6 h water abstention period. Endotracheal intubation was performed, and anesthesia was maintained with isoflurane (1.2–2.04% minimum alveolar concentration) throughout the endovascular procedure. Vital physiological parameters—including arterial blood pressure, heart rate, and end-tidal gas concentrations (oxygen and carbon dioxide)—were continuously monitored, with expired CO_2_ maintained between 30 and 35 mmHg. At the end of the procedure, euthanasia was carried out via intravenous administration of 20 mmol potassium chloride.

Heparin (5000 units) was administered intravenously as a bolus prior to vascular access. A 7F femoral sheath was inserted into the right femoral artery under ultrasound guidance (Sonoace7; Samsung, Seoul, Republic of Korea). A 7F Envoy guiding catheter (Codman Neurovascular, Raynham, MA, USA) was then navigated to the distal common carotid artery under single-plane angiography. Using a 0.014-inch exchange microwire (Transcend; Boston Scientific, Natick, MA, USA), both the modified cranial IV-OCT and conventional coronary OCT catheters were advanced into the distal segment of the external carotid artery (ECA).

To indirectly assess the trackability and pushability of the catheters in vivo, two parameters were analyzed: (1) the catheter tension angle (CTA) and (2) the pass of catheter (POC) through the final ECA curvature. The CTA was defined as the angle formed between a vertical reference line projected from the center of the horizontal parent artery and the distal shaft of the catheter, serving as an indicator of trackability. As both catheters successfully traversed the 1st, 2nd, and 3rd curves, measurements of CTA were taken between the 3rd and 4th curvatures, and POC was evaluated at the 4th curve. A larger CTA indicated greater catheter conformity and trackability within the vascular lumen. POC was defined as successful advancement of the catheter tip past the 4th curve with minimal resistance, whereas a non-pass referred to the catheter’s failure to proceed beyond this anatomical point despite applied force.

### 2.4. Experimental Protocol and Data Analysis

The mechanical properties—specifically, trackability (resistance) and pushability—of both modified and conventional coronary IV-OCT catheters were tested in a simulated vessel flow path. These two parameters were assessed using a total of 50 experiments, with 5 tests per catheter and 5 catheters of each type. To further evaluate catheter performance in vivo, CTA and POC were measured as indirect indicators of trackability and pushability, respectively, during advancement through the terminal curvature of the ECA in animal models. This in vivo assessment involved 72 trials, with 3 test repetitions conducted for each of 3 catheters per type across bilateral ECAs in two swine.

To minimize procedural bias and iatrogenic vessel injury or vasospasm—common in repeated catheterization protocols—the order of catheter testing was randomized and alternated between modified and conventional coronary IV-OCT devices across both ECAs. Each catheter type was tested non-consecutively, and the sequence was counterbalanced between animals and vessel sides to avoid order-dependent effects. Angiography was performed before and after each insertion to ensure vessel patency and to exclude any signs of mechanical trauma or spasm that could confound the evaluation of trackability, pushability, CTA, or POC.

The results are presented as the mean ± standard deviation and number (%). Statistical analysis was performed with SPSS software version 27 (IBM Corp., Armonk, NY, USA) using independent *t*-tests to evaluate the significance of the observed differences in trackability (N) and pushability (mm) between the two catheters (*p* < 0.05) in a simulated vessel flow path. In the animal models, independent sample *t*-tests were performed to analyze the differences in the CTA, and Fisher’s exact test was used to analyze the differences in the number of POC through the 4th curve (*p* < 0.05).

## 3. Results

### 3.1. Comparative Mechanical Performance of Modified and Conventional Coronary IV-OCT Catheters in Simulated Vessel Path

**Figure 2:**
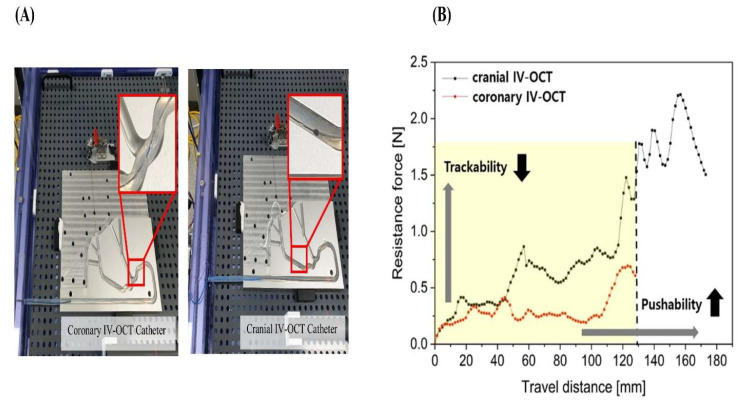
Evaluation of coronary and cranial IV-OCT catheters using a simulated vessel path. (**A**) Schematic of the simulated vessel path (ASTM F2394) used for benchtop testing of catheter mechanical performance and (**B**) Graph showing the results for the resistance and distance of coronary and cranial IV-OCT catheters along the simulated vessel path. The figure and graph were generated using the same experimental setup as our previous work [18], incorporating baseline mechanical data of the catheters from that study to ensure reference consistency and methodological alignment under the same ASTM F2394-based benchtop testing protocol.

Table 1(A) and Figure 2 present the comparative mechanical results of pushability and trackability between the modified cranial IV-OCT catheter and the conventional coronary IV-OCT catheter, evaluated using a standardized simulated blood-vessel flow path. The baseline data for the coronary IV-OCT catheter, previously reported in our earlier publication [18], were incorporated for methodological consistency and reference comparison, as both the previously developed bOCT catheter and the current cranial IV-OCT catheter were derived from the same conventional coronary OCT platform. Because the current design of cranial IV-OCT catheter involved only minor structural refinements—including extension of the non-braided distal segment and adjustment of the shaft stiffness gradient—the mechanical evaluation under identical conditions produced results that were identical to those previously reported. This similarity likely reflects that the refinements, though conceptually significant for intracranial compatibility, did not substantially modify the overall mechanical behavior under controlled benchtop testing.

To ensure scientific transparency and methodological continuity, the previously validated mechanical data were reused for the simulated-vessel-path comparison. This approach prevented unnecessary repetition of identical experiments while allowing for a precise and reproducible assessment of mechanical behavior between the two catheter designs. Under these standardized conditions, the modified IV-OCT catheter, incorporating a braided shaft, demonstrated superior pushability relative to its coronary counterpart. It achieved an average advancement distance of 172.9 ± 1.96 mm, significantly exceeding the conventional coronary IV-OCT catheter’s mean penetration of 127.9 ± 2.86 mm, corresponding to an approximate 35% improvement in pushability.

In terms of trackability, defined by the peak resistance encountered during advancement, the modified IV-OCT catheter exhibited a higher maximum resistance of 1.47 ± 0.036 N, in contrast to lower resistance of 0.69 ± 0.032 N for the conventional IV-OCT catheter. This increase in resistance is attributable to the structural modifications of the coronary IV-OCT catheter—specifically, the extended over-the-wire segment and proposed braided structure —which, while enhancing pushability, also contribute to greater frictional forces within the flow path.

### 3.2. In Vivo Performance of Modified and Conventional Coronary IV-OCT Catheters Using Animal Model

**Figure 3:**
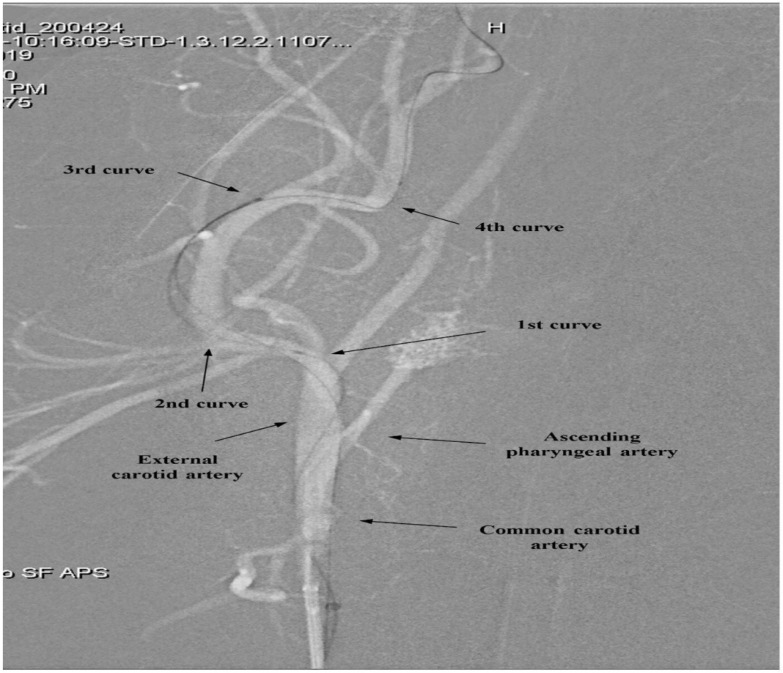
Segmentation of the external carotid artery (ECA) curvature for catheter evaluation.

**Figure 4:**
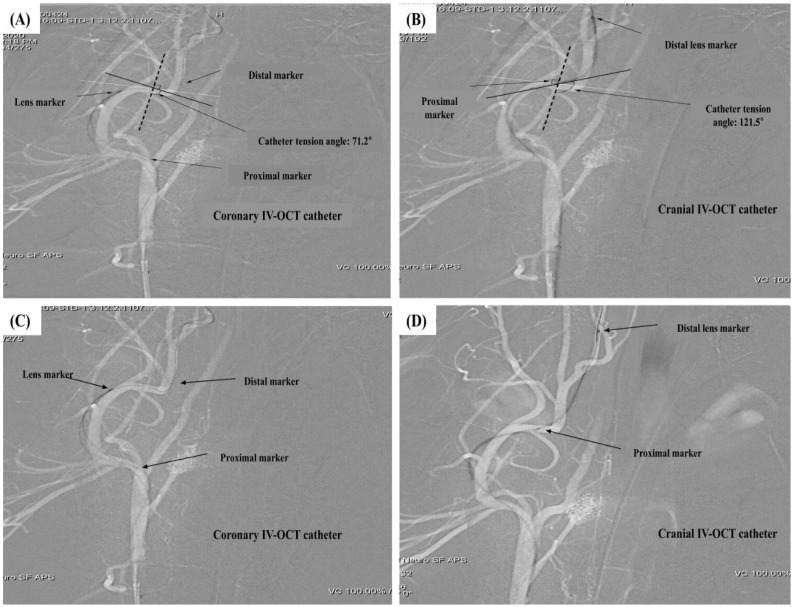
Preclinical evaluation of modified cranial IV-OCT catheter trackability and pushability in the external carotid artery (ECA). (**A**,**B**) Catheter tension angle (CTA) was used to assess trackability. CTA was defined as the angle between the vertical line (black dashed line) from the center of the parallel line to the horizontal parent artery (white line) and the distal catheter line (black line). Measured CTA values were 71.2° for the coronary IV-OCT catheter and 121.5° for the cranial IV-OCT catheter. (**C**,**D**) Pass of catheter (POC) was used to assess pushability. POC was defined as the distal advancement of the catheter beyond the 4th ECA curve. Non-pass was defined as no further distal movement. Coronary and cranial IV-OCT catheters showed non-passing and successful passing, respectively, of the distal catheter tip beyond the 4th curve.

Figure 3 illustrates the segmentation of the ECA into four curves (1st–4th), which served as reference points for evaluating catheter navigation. Table 1(B) and Figure 4 present the comparative in vivo evaluation of modified and conventional coronary IV-OCT catheters, using CTA and POC through the fourth curvature of the ECA as indirect metrics of trackability and pushability, respectively.

The modified IV-OCT catheter exhibited a significantly higher mean CTA of 115.8 ± 8.5°, compared to 77.6 ± 10.3° for the conventional coronary IV-OCT catheter (*p* < 0.05), indicating superior alignment and vessel wall conformity in tortuous anatomy. Similarly, the modified catheter exhibited a markedly higher success rate in traversing the final curvature, with a POC rate of 30 out of 36 trials (83.3%), as opposed to only 4 out of 36 (11.1%) for the conventional catheter (*p* < 0.05). Angiographic imaging further illustrated these differences: the conventional IV-OCT failed to advance beyond the final curvature, with a CTA of 71.2° and no observable distal tip movement (Figure 4A,C), while the modified IV-OCT achieved a higher CTA of 121.5° and successfully crossed the fourth curve (Figure 4B,D).

## 4. Discussion

Intravascular optical coherence tomography (IV-OCT), which represents a pivotal imaging modality in contemporary coronary diagnostics and therapeutic interventions, remains constrained in its translation to neurovascular applications due to the anatomical complexity of intracranial arteries [14,22]. Intracranial vessels differ markedly from coronary arteries; they exhibit sharply angulated trajectories, a lack of adventitia, are surrounded by minimal soft tissue, and are highly susceptible to perforation during endovascular device deliver. These structural challenges render conventional coronary IV-OCT systems—with their short over-the-wire segments and highly flexible shafts—mechanically inadequate for anterior circulation navigation. The short over-the-wire segment limits distal control and axial force transmission, while the overly flexible shaft cannot maintain pushability across sharp bifurcations. Moreover, the thin and fragile walls of intracranial vessels increase their susceptibility to perforation when devices buckle or whip due to inadequate force transmission. In addition, because intracranial arteries are encased within rigid cranial structures and surrounded by minimal soft tissue, they have limited capacity to deform, further amplifying the risk of vessel injury when using devices designed for coronary arteries.

This limitation is clinically significant, particularly in Asian, Black, and Hispanic populations, where the prevalence of intracranial atherosclerotic disease is disproportionately high compared to Western populations [23]. IV-OCT has the potential to deliver high-resolution intraluminal imaging for the assessment of plaque morphology, thrombus burden, and in-stent restenosis, thereby guiding appropriate selection between medical management and endovascular therapy [5,11,24]. To address these unmet clinical and mechanical needs, we developed a modified IV-OCT prototype incorporating two major design innovations: (1) an elongated 300 mm over-the-microwire segment to enhance axial force transmission, and (2) a partially braided catheter shaft to improve pushability while preserving OCT imaging quality by limiting metallic interference artifacts. However, because metallic braiding can introduce scattering and reflection artifacts that interfere with OCT signal acquisition and obscure the vessel wall, the distal 50 mm segment was intentionally left non-braided. This dual configuration ensured sufficient proximal support while preserving imaging quality at the distal tip.

In our previous study, we developed and characterized a brain-specific OCT catheter [18], which established the foundation for adapting coronary-type OCT systems to the neurovascular environment. That work focused primarily on the design, fabrication, and conceptual feasibility, demonstrating the structural modification required for safe navigation through tortuous cerebrovascular anatomy. Building upon this groundwork, the present study introduces a cranial IV-OCT catheter, a structurally refined version designed to enhance mechanical suitability for intracranial applications.

Both the previously developed bOCT and the current cranial IV-OCT catheters were derived from the same conventional coronary OCT platform. However, the aim of this study was not to compare the two prototypes but to evaluate the mechanical feasibility and in vivo applicability of the modified catheter. The coronary OCT catheter served as the baseline model to assess the degree of structural adaptation necessary to achieve neurovascular compatibility. Structural refinements—including the extension of the non-braided distal segment and optimization of the shaft stiffness gradient—were implemented to enhance pushability, trackability, and safety during intracranial navigation.

To ensure methodological continuity and scientific transparency, the mechanical data from the previous study were reused for the simulated-vessel-path comparison, as both investigations followed the same ASTM F2394-based benchtop testing setup under identical conditions. This reuse avoided unnecessary repetition while ensuring reproducibility and consistency in mechanical benchmarking. Although the refinements produced mechanical trends consistent with the earlier findings, they are crucial for improving catheter performance under physiological conditions, minimizing frictional stress and metallic interference, and enhancing navigational precision. Consequently, the novelty of this work lies in the structural refinement and preclinical validation of a coronary-derived IV-OCT system for intracranial applications, rather than in remeasurement of identical mechanical parameters.

However, in vitro testing using the simulated vascular path demonstrated a 35% improvement in pushability of the modified IV-OCT catheter (172.9 ± 1.96 mm vs. 127.9 ± 2.86 mm), validating the effectiveness of the structural modifications. The increase in maximum resistance observed in the proposed IV-OCT catheter (1.47 ± 0.036 N) compared to the conventional coronary IV-OCT (0.69 ± 0.032 N) was an anticipated trade-off due to the increased rigidity associated with braided shaft. Previous studies have reported similar observations where mechanical reinforcement improves pushability at the cost of elevated frictional resistance [25]. Moreover, the applied hydrophilic coating effectively mitigated friction during advancement.

The animal model further validated the modified catheter’s superiority, particularly in traversing the fourth curvature of the ECA, a region modeled to replicate intracranial tortuosity. Direct measurement of trackability, as performed in simulated models, was impractical; thus, we performed in vivo validation in a swine model using CTA and POC as indirect yet functionally relevant metrics for trackability and pushability. The modified IV-OCT achieved significantly higher CTA values (115.8 ± 8.5° vs. 77.6 ± 10.3°, *p* < 0.05) and greater POC rates (83.3% vs. 11.1%, *p* < 0.05), indicating superior navigational performance across the 4th vascular curvature. Angiographic imaging supported these findings, with the modified IV-OCT advancing beyond the tortuous segment where the conventional catheter failed.

Taken together, these results highlight that ‘trackability’ was evaluated differently in the two models: direct resistance in the benchtop path versus functional navigation performance in vivo. While the benchtop data indicated greater resistance for the modified catheter, the in vivo outcomes demonstrated superior navigability in tortuous vessels. This distinction underscores that the benchtop model primarily quantifies mechanical friction in a standardized environment, whereas the in vivo model better reflects clinically meaningful performance. Accordingly, the in vivo findings provide stronger evidence of the catheter’s suitability for neurovascular applications.

Beyond mechanical considerations, an essential aspect of the design was ensuring that the modified catheter could interface effectively with existing IV-OCT platforms and support reliable image acquisition. The elimination of the septum separating the microwire lumen from the OCT optical core reduced the overall catheter diameter and facilitated smoother optical core alignment, thereby enhancing compatibility with standard IV-OCT delivery systems. Additionally, the distal 50 mm segment was intentionally left non-braided to minimize metallic interference and preserve imaging quality, while the proximal shaft retained a braided configuration to provide sufficient pushability and mechanical stability. Combined with the elongated over-the-wire segment, these structural modifications are expected to improve catheter positioning, enhance imaging stability, and enable more precise lesion targeting in future benchtop and in vivo OCT imaging studies.

Despite the success in demonstrating the mechanical superiority of the modified IV-OCT catheter, this study has several limitations. First, the current prototype lacks optical validation; hence, no OCT images were acquired. While the mechanical feasibility has been confirmed, future iterations of this work will focus on integrating the optical imaging components—such as the gradient-index (GRIN) lens, fiber-optic core, and rotational scanning assembly—into the catheter system to ensure full functionality with existing IV-OCT consoles. Subsequent studies will include benchtop and in vivo experiments to validate imaging performance, assess image quality in tortuous intracranial vessels, and confirm the catheter’s capability for precise lesion visualization and diagnostic use in neurovascular interventions. Second, due to the limited number of animals, each ECA underwent multiple catheterizations, which may introduce bias due to progressive vasospasm or vessel trauma. However, randomization of catheter testing order and real-time fluoroscopic assessment helped mitigate these confounding effects. Additionally, while the benchtop and animal models provided consistent and controlled environments for evaluating catheter mechanics, they do not fully replicate the smaller caliber, sharp angulations, and complex tortuosity of human intracranial vessels—particularly within the anterior circulation. These challenges are most pronounced at distal segments such as the MCA and ACA bifurcations. To address this, future studies will evaluate the catheter’s performance in more anatomically relevant models, including 3D-printed patient-derived phantoms, higher-tortuosity ex vivo platforms, and alternative animal models, while further refining the design to enhance distal flexibility and adaptability in highly tortuous pathways. The conventional coronary IV-OCT catheter was selected as a comparator to directly evaluate mechanical improvements based on its known structural limitations; however, future studies comparing the modified IV-OCT catheter with neurovascular delivery catheters will be important to contextualize its performance in clinical neurointerventional applications.

## 5. Conclusions

In summary, this study presents evidence supporting the mechanical viability of a modified cranial IV-OCT catheter designed for neurovascular applications. These advancements, particularly in pushability and trackability, mark a significant step toward enabling OCT-guided interventions in intracranial neurovascular procedures. Future work will focus on integrating imaging capabilities and validating clinical utility in more diverse vascular environments.

## Figures and Tables

**Table 1:** Statistical analysis of coronary vs. cranial IV-OCT catheter performance: Resistance and distance in simulated path (A) and CTA (°) and POC through the 4th curve of the ECA in animal Model (B).

Variables		Coronary	Cranial	* *p* Value
Simulated Path (A)	Resistance (gram force, N)	0.69 ± 0.032	1.47 ± 0.036	*p* < 0.05
	Distance (mm)	127.9 ± 2.86	172.9 ± 1.96	*p* < 0.05
Animal model (B)	Catheter tension angle (CTA) (°)	77.6 ± 10.3	115.8 ± 8.5	*p* < 0.05
	Pass of catheter (POC) through 4th curve of ECA (%)			
	Pass	4 (11.1%)	30 (83.3%)	*p* < 0.05
	Non-pass	32 (88.9%)	6 (16.7%)	

The values are presented as the mean ± standard deviations and numbers (%). * = *p* ≤ 0.05.

## Data Availability

Data are contained within the article.

## Supplementary Materials

The following supporting information can be downloaded at: https://www.mdpi.com/article/10.3390/bios15110755/s1, Figure S1: Schematic illustration showing the whole structure of modified cranial IV-OCT with the L, OD, ID, and each segment of this catheter, including 300 mm over-the-wire from the distal end entry to the proximal side exit port. (a) Distal nonbraided OCT lens part (50 mm). (b) Distal braided shaft part (250 mm). (c) Proximal braided part (1130 mm). (d) Proximal braided shaft part (135 mm). (e) Distal end entry port. (f) Proximal side exit port. L, length; OD, outer diameter; ID, inner diameter. The schematic was redrawn following the configuration style of our previous publication [18], with updated OD/ID dimensions and an extended non-braided segment (50 mm) reflecting the refined cranial IV-OCT catheter design; Figure S2: Prototype cranial IV-OCT. (a) Whole catheter. (b) Microwire through the distal end entry port. (c) Microwire through the proximal side exit port. The prototype image follows the same configuration style as our previous publication [18] and is included here to provide a visual overview of the catheter’s overall assembly. No new structural modifications are depicted in this figure, as the design refinements are described in the main text; Table S1: Differences in structural characteristics between conventional coronary and modified cranial IV-OCT catheters.

## Abbreviations

The following abbreviations are used in this manuscript:

IV-OCT	Intravascular optical coherence tomography
CTA	Catheter tension angle
POC	Pass of catheter
ECA	External carotid artery

## References

[1] van der Marel K., Gounis M.J., Weaver J.P., de Korte A.M., King R.M., Arends J.M., Brooks O.W., Wakhloo A.K., Puri A.S. (2016). Grading of Regional Apposition after Flow-Diverter Treatment (GRAFT): A comparative evaluation of VasoCT and intravascular OCT. J. Neurointerv. Surg..

[2] Chen C.-J., Kumar J.S., Chen S.H., Ding D., Buell T.J., Sur S., Ironside N., Luther E., Ragosta M., Park M.S. (2018). Optical coherence tomography: Future applications in cerebrovascular imaging. Stroke.

[3] Yun S.H., Tearney G.J., Vakoc B.J., Shishkov M., Oh W.Y., Desjardins A.E., Suter M.J., Chan R.C., Evans J.A., Jang I.-K. (2006). Comprehensive volumetric optical microscopy in vivo. Nat. Med..

[4] Tearney G.J., Regar E., Akasaka T., Adriaenssens T., Barlis P., Bezerra H.G., Bouma B., Bruining N., Cho J.-m., Chowdhary S. (2012). Consensus standards for acquisition, measurement, and reporting of intravascular optical coherence tomography studies: A report from the International Working Group for Intravascular Optical Coherence Tomography Standardization and Validation. J. Am. Coll. Cardiol..

[5] Ughi G.J., Wang H., Gerbaud E., Gardecki J.A., Fard A.M., Hamidi E., Vacas-Jacques P., Rosenberg M., Jaffer F.A., Tearney G.J. (2016). Clinical characterization of coronary atherosclerosis with dual-modality OCT and near-infrared autofluorescence imaging. JACC. Cardiovasc. Imaging..

[6] Nam H.S., Kim C.S., Lee J.J., Song J.W., Kim J.W., Yoo H. (2016). Automated detection of vessel lumen and stent struts in intravascular optical coherence tomography to evaluate stent apposition and neointimal coverage. Med. Phys..

[7] Ughi G.J., Adriaenssens T., Onsea K., Kayaert P., Dubois C., Sinnaeve P., Coosemans M., Desmet W., D’hooge J. (2012). Automatic segmentation of in-vivo intra-coronary optical coherence tomography images to assess stent strut apposition and coverage. Int. J. Cardiovasc. Imaging..

[8] Lopes D.K., Johnson A.K. (2012). Evaluation of cerebral artery perforators and the pipeline embolization device using optical coherence tomography. J. Neurointerv. Surg..

[9] Given C.A., Ramsey C.N., Attizzani G.F., Jones M.R., Brooks W.H., Bezerra H.G., Costa M.A. (2015). Optical coherence tomography of the intracranial vasculature and Wingspan stent in a patient. J. Neurointerv. Surg..

[10] Chang H., Yoon H.J., Hong J.-H., Kim C.-H., Sohn S.-i., Lee C.-Y. (2016). Republished: A lotus root-like appearance in carotid stenosis on optical coherence tomography. J. Neurointerv. Surg..

[11] Xu X., Li M., Liu R., Yin Q., Shi X., Wang F., Gao J., Xu G., Ye R., Liu X. (2020). Optical coherence tomography evaluation of vertebrobasilar artery stenosis: Case series and literature review. J. Neurointerv. Surg..

[12] Yan L., Dmytriw A.A., Yang B., Jiao L. (2021). Optical coherence tomography of plaque erosion and thrombus in severe vertebral artery stenosis. Diagnostics.

[13] Dohad S., Zhu A., Krishnan S., Wang F., Wang S., Cox J., Henry T.D. (2018). Optical coherence tomography guided carotid artery stent procedure: Technique and potential applications. Catheter. Cardiovasc. Interv..

[14] Gounis M.J., Ughi G.J., Marosfoi M., Lopes D.K., Fiorella D., Bezerra H.G., Liang C.W., Puri A.S. (2019). Intravascular optical coherence tomography for neurointerventional surgery. Stroke.

[15] Pereira V.M., Lylyk P., Cancelliere N., Lylyk P.N., Lylyk I., Anagnostakou V., Bleise C., Nishi H., Epshtein M., King R.M. (2024). Volumetric microscopy of cerebral arteries with a miniaturized optical coherence tomography imaging probe. Sci. Transl. Med..

[16] Gao P., Gui L., Yang B., Krings T., Jiao L. (2018). Optical coherence tomography of spontaneous basilar artery dissection in a patient with acute ischemic stroke. Front. Neurol..

[17] Li D., Tang T., Hu T., Yu C.-Y., Thomas A.M., Zhao M.-H., Fan T.-P., Li S. (2023). Teaching neuroImage: Etiologic investigation using optical coherence tomography during thrombectomy. Neurology.

[18] Jung T.-M., Nairuz T., Kim C.-H., Lee J.-H. (2025). Development of a Brain Catheter for Optical Coherence Tomography in Advanced Cerebrovascular Diagnostics. Biosensors.

[19] Mathews M.S., Su J., Heidari E., Levy E.I., Linskey M.E., Chen Z. (2011). Neuroendovascular optical coherence tomography imaging and histological analysis. Neurosurgery.

[20] Ughi G.J., Marosfoi M.G., King R.M., Caroff J., Peterson L.M., Duncan B.H., Langan E.T., Collins A., Leporati A., Rousselle S. (2020). A neurovascular high-frequency optical coherence tomography system enables in situ cerebrovascular volumetric microscopy. Nat. Commun..

[21] (2022). Standard Guide for Measuring Securement of Balloon-Expandable Vascular Stent Mounted on Delivery System.

[22] Prati F., Guagliumi G., Mintz G.S., Costa M., Regar E., Akasaka T., Barlis P., Tearney G.J., Jang I.-K., Arbustini E. (2012). Expert review document part 2: Methodology, terminology and clinical applications of optical coherence tomography for the assessment of interventional procedures. Eur. Heart. J..

[23] Bang O.Y. (2014). Intracranial atherosclerosis: Current understanding and perspectives. J. Stroke..

[24] Xu R., Zhao Q., Wang T., Yang Y., Luo J., Zhang X., Feng Y., Ma Y., Dmytriw A.A., Yang G. (2023). Optical coherence tomography in cerebrovascular disease: Open up new horizons. Transl. Stroke. Res..

[25] Schmidt W., Lanzer P., Behrens P., Topoleski L., Schmitz K.P. (2009). A comparison of the mechanical performance characteristics of seven drug-eluting stent systems. Catheter. Cardiovasc. Interv..

