# Supplementary material for: Design Optimization and Mechanical Performance Evaluation of a Modified Coronary IV-OCT Catheter Adapted for Intracranial Navigation: A Preclinical Study
Source: Biosensors (Basel). 2025 Nov 12;15(11):755. doi: 10.3390/bios15110755 (PMC12650042; doi:10.3390/bios15110755)
Supplement: Supplementary file 1 [file biosensors-15-00755-s001.zip › biosensors-3853076-supplementary.pdf]

## Supplemental Materials

# Design Optimization and Mechanical Performance Evaluation of a Modified Coronary IV-OCT Catheter Adapted for Intracranial Navigation: A Preclinical Study

## Manufacturing of the modified cranial IV-OCT catheter

The modified cranial IV-OCT catheter developed for this study consisted of a shaft and a manifold handle. The shaft was divided into 4 segments from the distal to proximal parts, and the specifications of the catheters are shown in supplementary Figure 1.

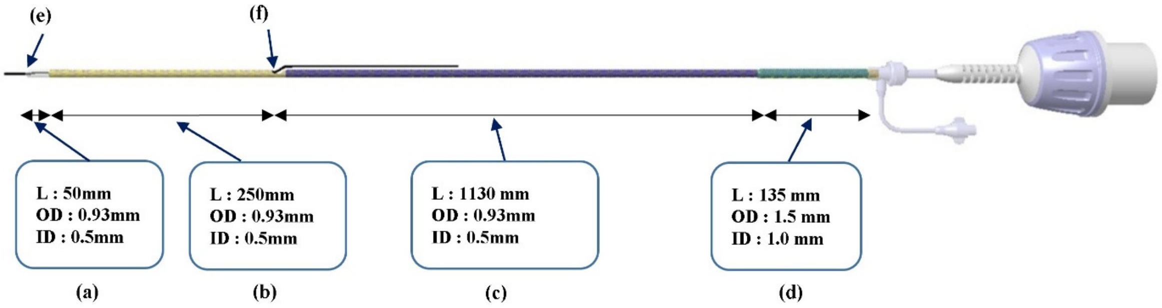


**Figure S1.** Schematic illustration showing the whole structure of modified cranial IV-OCT with the L, OD, ID, and each segment of this catheter, including 300 mm over-the-wire from the distal end entry to the proximal side exit port. (a) Distal nonbraided OCT lens part (50 mm). (b) Distal braided shaft part (250 mm). (c) Proximal braided part (1130 mm). (d)Proximal braided shaft part (135 mm). (e) Distal end entry port. (f) Proximal side exit port. L, length; OD, outer diameter; ID, inner diameter. The schematic was redrawn following the configuration style of our previous publication [18], with updated OD/ID dimensions and an extended non-braided segment (50 mm) reflecting the refined cranial IV-OCT catheter design.

(1) Distal part: The distal nonbraided OCT lens part was designed to allow both the microwire and the OCT lens to pass through a single lumen, thereby reducing the overall profile of the catheter's distal end. The distal braided shaft was composed of an outer jacket (Pebax 5533 SA 01 MED, Foster, Putnam, CT, USA), braided wire (SUS304V, 0.01×0.05 mm, Ulbrich, Inc., North Haven, CT, USA) and polytetrafluoroethylene (PTFE) liner (ZEUS., Inc., Orangeburg, SC, USA) for enhanced pushability. In particular, the innermost PTFE liner, which had a low coefficient of friction, also helped stably transport the coil with an optical mirror.

(2) Proximal part: The proximal part, like the distal part, was developed with a three-layer braided structure. Considering the need for greater pushability in comparison to the distal part, the section closer to the handle was designed with a larger outer diameter. Furthermore, a harder outer jacket consisting of a medical-grade polymer (Pebax 7233 SA 01 MED, Foster, Putnam, CT, USA) was employed to enhance the overall rigidity of the proximal part.

(3) Distal end entry and proximal side exit port: The distal end entry and proximal side exit port of the cranial IV-OCT catheter were configured to allow passage of a 0.014-inch microwire.

(4) Hydrophilic coating of the catheter shaft: The shaft surface of the cranial IV-OCT catheter was coated with a hyaluronic acid-based hydrophilic coating (Noacoat_base, Noanix, South Korea) for enhanced trackability.

The cranial IV-OCT prototype developed according to the abovementioned specifications, was shown in supplementary Figure 2. While the visual configuration remains consistent with the design shown in our previous publication [18], this figure is provided solely to give contextual understanding of the catheter structure employed in this extended work, which focuses on mechanical and preclinical evaluation rather than on new fabrication.

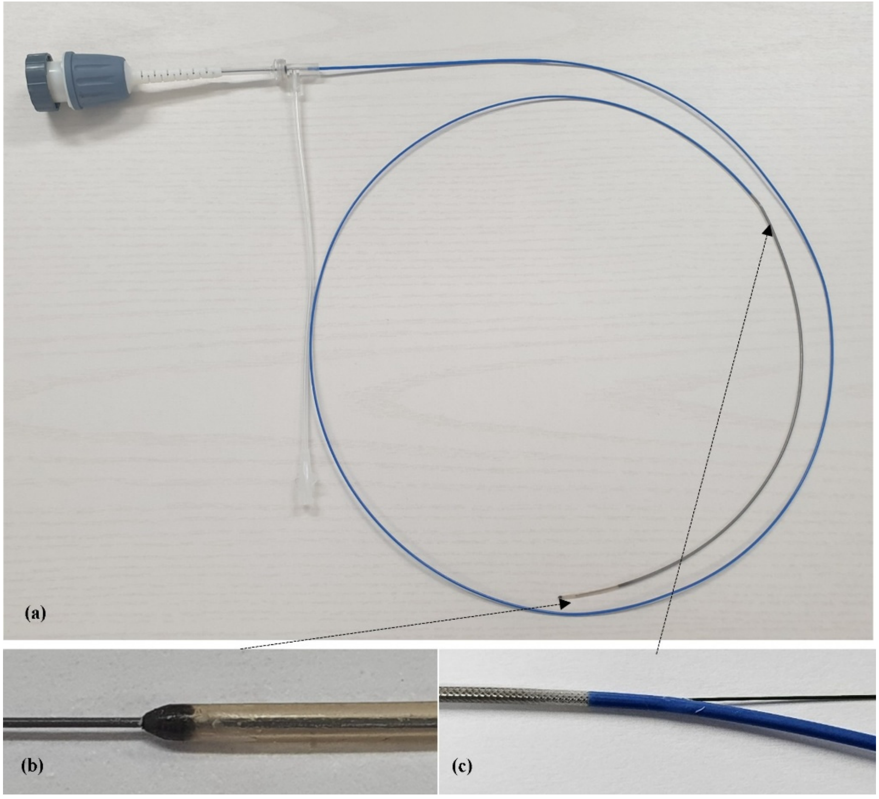


**Figure S2.** Prototype cranial IV-OCT. (a) Whole catheter. (b) Microwire through the distal end entry port. (c) Microwire through the proximal side exit port. The prototype image follows the same configuration style as our previous publication [18] and is included here to provide a visual overview of the catheter's overall assembly. No new structural modifications are depicted in this figure, as the design refinements are described in the main text.

Moreover, the structural differences between coronary and cranial IV-OCT are listed in supplementary Table 1.

**Table S1.** Differences in structural characteristics between conventional coronary and modified cranial IV-OCT catheters

| Variables | Coronary IV-OCT catheter | Cranial IV-OCT catheter |
|---|---|---|
| Length of over-the-wire portion from distal end entry to proximal side exit port | 20 mm | 300 mm |
| Location of distal end of OCT lens | Just behind proximal side hole of 20 mm distal tip | Just behind distal end of nonbraided portion, not beyond distal catheter end |
| Braided segment | Nonbraided | All braided except distal nonbraided OCT lens portion of 50 mm distal tip |
| Hydrophilic coating | yes | yes |

IV-OCT: intravascular optical coherence tomography

## References

18. Jung, T.-M.; Nairuz, T.; Kim, C.-H.; Lee, J.-H. Development of a Brain Catheter for Optical Coherence Tomography in Advanced Cerebrovascular Diagnostics. Biosensors 2025, 15, 170.
